# Bioinformatic Analyses Identify a Prognostic Autophagy-Related Long Non-coding RNA Signature Associated With Immune Microenvironment in Diffuse Gliomas

**DOI:** 10.3389/fcell.2021.694633

**Published:** 2021-06-15

**Authors:** Shengchao Xu, Lu Tang, Zhixiong Liu, Kui Yang, Quan Cheng

**Affiliations:** ^1^Department of Neurosurgery, Xiangya Hospital, Central South University, Changsha, China; ^2^Department of Thoracic Surgery, Xiangya Hospital, Central South University, Changsha, China; ^3^National Clinical Research Center for Geriatric Disorders, Xiangya Hospital, Central South University, Changsha, China

**Keywords:** diffuse glioma, autophagy, long non-coding RNA, immune microenvironment, signature

## Abstract

**Background:**

Autophagy and long non-coding RNA (lncRNA) play a critical role in tumor progression and microenvironment. However, the role of autophagy-related lncRNAs (ARLs) in glioma microenvironment remains unclear.

**Methods:**

A total of 988 diffuse glioma samples were extracted from TCGA and CGGA databases. Consensus clustering was applied to reveal different subgroups of diffuse gliomas. Kaplan-Meier analysis was used to evaluate survival differences between groups. The infiltration of immune cells was estimated by ssGSEA, TIMER, and CIBERSORT algorithms. The construction of ARL signature was conducted using principal component analysis.

**Results:**

Consensus clustering revealed two clusters of diffuse gliomas, in which cluster 1 was associated with poor prognosis and enriched with malignant subtypes of gliomas. Moreover, cluster 1 exhibited high apoptotic and immune characteristics, and it had a low purity and high infiltration of several immune cells. The constructed ARL signature showed a promising accuracy in predicting the prognosis of glioma patients. ARL score was significantly elevated in the malignant subtype of glioma and the high ARL score indicated a poor prognosis. Besides, the high ARL score notably indicated low tumor purity and high infiltration of macrophages and neutrophils.

**Conclusion:**

Our study developed and validated a novel ARL signature for the classification of diffuse glioma, which was closely associated with glioma immune microenvironment and could serve as a promising prognostic biomarker for glioma patients.

## Introduction

Gliomas are the most common type of primary cancer in the central nervous system (Ostrom et al., 2019). In adults, diffuse glioma is termed as a variety of brain cancers including WHO grade II to grade IV tumors. Typically, grade IV glioma is the most malignant subtype compared with grade II and III gliomas. The median overall survival (OS) of grade II, grade III, and grade IV gliomas is 78.1, 37.6, and 14.4 months, respectively (Yang et al., 2013). Several biomarkers are found to be associated with the prognosis of glioma patients. In different grades of glioma, patients with isocitrate dehydrogenase 1 (IDH1) mutant glioma had significant prolonged OS compared with *IDH1* wildtype ones (Yan et al., 2009; Weller et al., 2015). Besides, 1p19q codeletion is considered as a favorable prognostic factor for glioma patients (Kaloshi et al., 2007; Eckel-Passow et al., 2015). Moreover, O6-methylguanine–DNA methyltransferase (MGMT) promoter methylation in gliomas indicates benefits from temozolomide treatment (Hegi et al., 2005; Everhard et al., 2006). Additionally, age is considered as a prognostic factor for glioma patients, and the younger age indicates a favorable prognosis (Li et al., 2009; Lin et al., 2020). However, although current management for gliomas has achieved great progress, the prognosis of glioma patients remains unfavorable because of tumor recurrence and chemoresistance. Therefore, there is a clear urgent to find potential targets for glioma treatment.

Tumor microenvironment is reported to play a crucial role in the development and progression of cancers. Typically, tumor microenvironment is consisted of stromal cells, extracellular matrix, and immune cells, in which infiltrated immune cells are demonstrated to correlated with the prognosis of various cancers (Jia et al., 2018; Shen et al., 2019; Huang et al., 2020). Although the central nervous system has long been considered as “immune privileged,” the discovery of lymphatic vessels along with the dural sinuses overturns this viewpoint (Louveau et al., 2015). Immune cells such as tumor-associated macrophages (TAMs) and regulatory T cells (Treg) could infiltrate into the brain and form an immunosuppressive microenvironment to facilitate the progression of glioma cells (Lim et al., 2018; Xu et al., 2020b). The exploration of glioma microenvironment would provide a better understanding for the occurrence and development of gliomas.

Autophagy is a catabolic process triggered by diverse signals and cellular stresses to remove unnecessary or dysfunctional components (Klionsky, 2008). Dysregulated autophagy is involved in multiple diseases including diabetes, infectious disease, and cancer (Chen et al., 2014; Sarparanta et al., 2017; Onorati et al., 2018). Huang et al. (2010) reported that decreased expression of autophagy-related genes, LC3B and Beclin 1, was associated with poor prognosis of glioblastoma patients. Besides, *SH3GLB1* and *MAPK8IP1* were correlated with autophagy and the OS of glioma patients (Zhang H. et al., 2017). Meanwhile, autophagy inhibitors such as chloroquine (CQ) and hydroxychloroquine (HCQ) appear to be promising therapeutics in cancer therapy (Onorati et al., 2018; Amaravadi et al., 2019). However, their applications should be tailored in specific situations because autophagy plays a context-dependent role in cancer and inappropriate utilization can be detrimental (Levy et al., 2017). Therefore, a better understanding of autophagy in the context of cancer would facilitate the translation of autophagy therapies in cancer treatment.

Long non-coding RNA (lncRNA) play a critical role in physiological and pathological processes (Mercer et al., 2009). Aberrant expression of lncRNA was associated with the grade, histological subtype, and prognosis of glioma patients (Jing et al., 2016; Wang et al., 2016; Peng et al., 2018). Besides, lncRNA is associated with the proliferation, progression, invasion, and prognosis of gliomas by exerting diverse biological activities (Deng et al., 2020; Liu C. et al., 2020; Ruan et al., 2020; Xin et al., 2020). Therefore, the exploration of novel lncRNAs as biomarkers in glioma microenvironment will provide novel insights into the development and progression of gliomas.

A previous study indicated that autophagy-related lncRNAs (ARLs) were associated with the prognosis of gliomas (Luan et al., 2019). However, their roles in glioma immune microenvironment remain unclear. Herein, our study extracted data from The Cancer Genome Atlas (TCGA) and Chinese Glioma Genome Atlas (CGGA) databases and adopted multiple algorithms, aiming to explore the prognostic value of ARLs in diffuse glioma and their correlation in glioma immune microenvironment.

## Materials and Methods

### Data Extraction

A total of 988 diffuse glioma samples were included in our study. The RNA-seq and clinical data were extracted from TCGA^1^ and CGGA^2^ databases. A total of 14,488 and 13,895 lncRNAs were identified in the TCGA and CGGA datasets, respectively. Characteristics of glioma samples in the TCGA (*n* = 672) and CGGA (*n* = 316) datasets were list in Table 1.

**TABLE 1:** Characteristics of TCGA and CGGA datasets.

	TCGA (*n* = 672)	CGGA (*n* = 316)
**Age**		
≤45	343	197
>45	329	119
**Gender**		
Male	387	196
Female	285	120
**Grade**		
II	256	102
III	265	76
IV	150	134
NA	1	4
**IDH1 status**		
Mutant	434	171
Wildtype	228	145
NA	10	0
**1p/19q status**		
Codel	171	67
Non-codel	497	246
NA	4	3
**MGMT status**		
Methylated	478	153
Unmethylated	157	144
NA	37	19

*IDH1, isocitrate dehydrogenase; MGMT, O6-methylguanine-DNA methyltransferase.*

### Identification of ARLs

The 231 autophagy-related genes were extracted from Human Autophagy Database^3^. In the TCGA and CGGA datasets, 218 and 205 autophagy-related genes were identified, respectively. To identify ARLs, Pearson correlation analysis was conducted between autophagy-related genes and all lncRNAs. Those with | R| > 0.5 and *p* < 0.01 were selected as ARLs.

### Consensus Clustering

In this study, “ConsensusClusterPlus” R package was used to cluster glioma samples based on the expression of ARLs. The clustering algorithm was partitioning around medoids and the distance was measured by the euclidean metric.

### Gene Set Variation Analysis (GSVA)

GSVA analysis was conducted to quantify the involvement of biological processes of each sample using “GSVA” R package (Hanzelmann et al., 2013).

### Gene Set Enrichment Analysis (GSEA)

Differentially expressed genes between cluster 1 and cluster 2 or high-score and low-score groups were screened, and those with false discovery rate (FDR) ≤ 0.05 were selected. The enrichment analyses were conducted using “clusterprofiler” R package including Hallmark and Gene Ontology (GO) gene sets.

### Immune Microenvironment Assessment

Estimation of Stromal and Immune cells in Malignant Tumor tissues using Expression data (ESTIMATE) analysis was conducted to calculate the stromal score, immune score, ESTIMATE score, and tumor purity of each sample using “estimate” R package (Yoshihara et al., 2013). Single-sample Gene Set Enrichment Analysis (ssGSEA), Tumor Immune Estimation Resource (TIMER), and CIBERSORT algorithms were adopted to evaluate the abundance of infiltrated immune cells in each sample (Barbie et al., 2009; Newman et al., 2015; Li et al., 2020).

### Construction of ARL Signature

With the application of Least Absolute Shrinkage and Selection Operator (LASSO) analysis, 38 ARLs were selected for the analysis. The construction of ARL signature was performed based on principal component analysis (PCA). Univariate Cox analysis identified 11 protective lncRNAs (hazard ratio < 1) and 27 risk lncRNAs (hazard ratio > 1). The ARL score was calculated according to the following algorithm: ARL score = Σ[(PC1 + PC2) × expression_*risk*_ - (PC1 + PC2) × expression_*pro*_]; where “risk” stood for risk genes and “pro” stood for protective genes.

### Subgroup Analysis

For subgroup analysis, diffuse glioma patients were divided into different groups based on features as follows: grades (grade II, III, or IV), age (≤45 years old or >45 years old), and *IDH1* status (wildtype or mutant).

### Statistical Analysis

Statistical analyses and visualization were performed using GraphPad Prism 8.0.1 and R 3.6.0. The Chi-square test was used to evaluate the difference of clinical features in different groups. Student’s *t*-test and one-way ANOVA analysis were used to estimate the differences between two groups and more than two groups. Kaplan-Meier analysis was for survival analysis between two groups of patients. Multivariate Cox analysis was used to evaluate the prognostic value of the ARL score. Time-dependent receiver operating characteristic (ROC) curve analysis was adopted to estimate the predictive value of the ARL score. Two-sided *p* ≤ 0.05 was regarded as statistically significant.

## Results

### Identification and Consensus Clustering of ARLs

**FIGURE 1:**
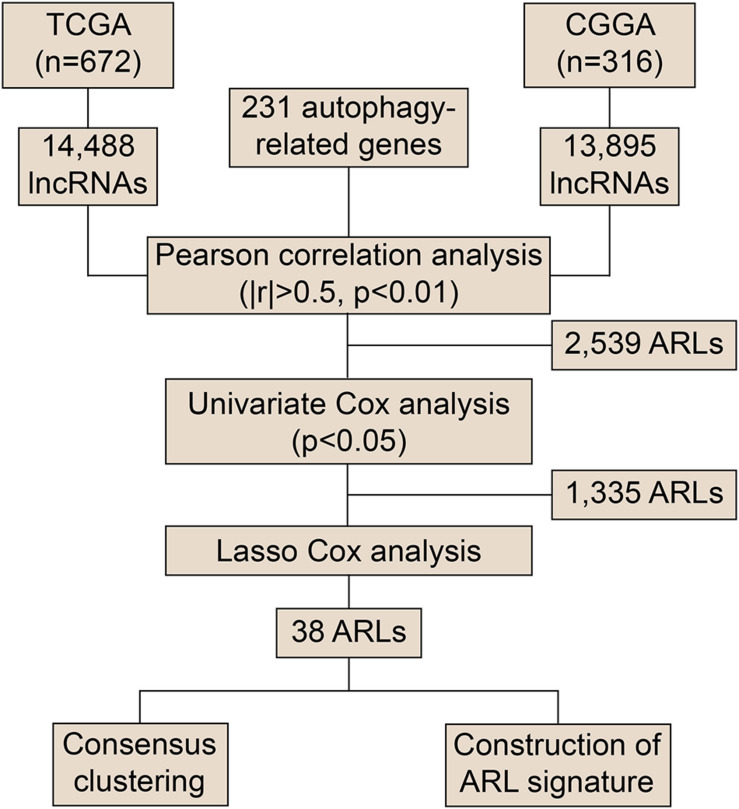
Flow chart of screening ARLs.

**FIGURE 2:**
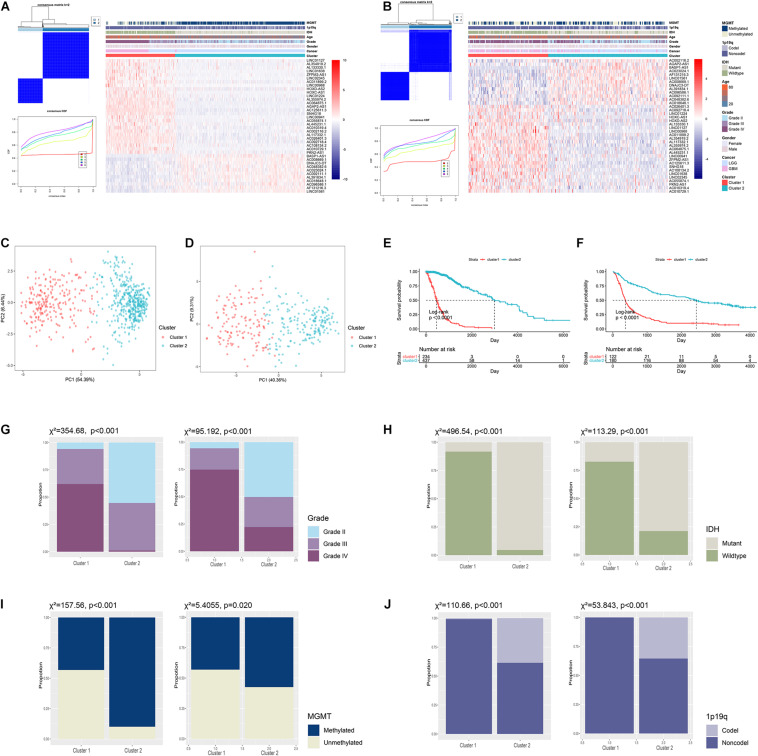
Unsupervised clustering of diffuse gliomas based on expression of ARLs. **(A,B)** Consensus clustering classified diffuse gliomas into two clusters in TCGA **(A)** and CGGA **(B)** datasets. **(C,D)** Principal component analysis of two clusters in TCGA **(C)** and CGGA **(D)** datasets. **(E,F)** Kaplan-Meier analysis of glioma patients in two clusters in TCGA **(E)** and CGGA **(F)** datasets. **(G–J)** The proportion of different grades **(G)**, IDH1 status **(H)**, MGMT status **(I)**, and 1p19q status **(J)** of gliomas in cluster 1 and cluster 2 in TCGA and CGGA datasets.

To explore the role of ARLs in gliomas, we extracted data from the TCGA and CGGA datasets. Pearson correlation analysis was conducted to screened potential ARLs in two datasets, in which 2,539 lncRNAs with | R| > 0.5 and *p* < 0.01 were selected (Figure 1 and Supplementary Table 1). Then 1,335 prognostic lncRNAs were picked using univariate cox analysis in the TCGA and CGGA datasets (Supplementary Table 2). Finally, we performed LASSO analysis to identify 38 ARLs for further analysis (Supplementary Figure 1 and Supplementary Table 3). Kaplan-Meier analysis revealed that randomly selected eight of 38 ARLs were significantly associated with the prognosis of glioma patients (*p* < 0.05) (Supplementary Figure 2). Consensus clustering was independently conducted in the TCGA and CGGA datasets, in which glioma patients were classified into two clusters (Figures 2A,B). As shown in the heatmap, 11 protective ARLs (hazard ratio < 1) were highly expressed in cluster 2, whereas 27 risk ARLs (hazard ratio < 1) were highly expressed in cluster 1 (Figures 2A,B and Supplementary Table 4). PCA analysis indicated that the two clusters were well classified (Figures 2C,D). Besides, patients in cluster 2 had more favorable prognoses than those in cluster 1 (*p* < 0.05) (Figures 2E,F). In cluster 1, there was a higher proportion of high-grade glioma (grade III and IV) than cluster 2 (*p* < 0.05) (Figure 2G). Besides, *IDH1* mutant glioma samples were enriched in cluster 2 (*p* < 0.05) (Figure 2H). Similarly, MGMT methylated glioma samples were enriched in cluster 2 (*p* < 0.05) (Figure 2I). Moreover, almost all 1p19q co-deleted glioma samples were in cluster 2 (*p* < 0.05; Figure 2J). With the conduct of unsupervised clustering, we identified two distinct subtypes of gliomas based on the expression of ARLs, in which cluster 1 had a high proportion of the malignant type of glioma and was associated with poor prognosis.

### Apoptotic and Immune Processes Were Enriched in Cluster 1

**FIGURE 3:**
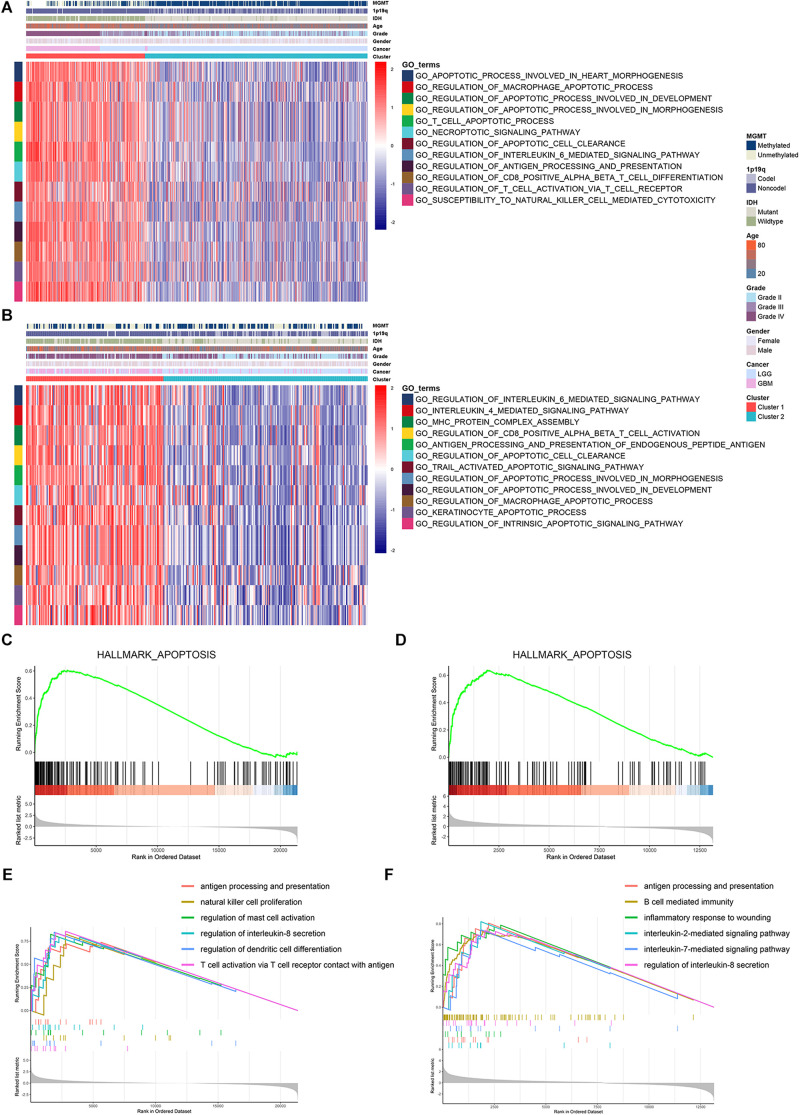
Cluster 1 was enriched with apoptotic and immune processes. **(A,B)** GSVA analysis revealed the enrichment of apoptotic and immune pathways in cluster 1 and cluster 2 in TCGA **(A)** and CGGA **(B)** datasets. **(C,D)** GSEA analysis of hallmark gene sets regarding differentially expressed genes between cluster 1 and cluster 2 in TCGA **(C)** and CGGA **(D)** datasets. **(E,F)** SEA analysis of differentially expressed genes between cluster 1 and cluster 2 in TCGA **(E)** and CGGA **(F)** datasets.

Then, we conducted GSVA analysis to further explore the characteristics between the two clusters. We found that apoptotic pathways such as necroptotic signaling pathway, macrophage apoptotic process, and T cell apoptotic process were highly enriched in cluster 1 (Figures 3A,B). Moreover, immune processes such as interleukin mediated signaling pathway, antigen processing and presentation, and T cell activation were also enriched in cluster 1 (Figures 3A,B). Further, we screened differentially expressed genes between cluster 1 and cluster 2 (Supplementary Figure 3 and Supplementary Table 5). GSEA analysis showed that genes highly expressed in cluster 1 were enriched in cell apoptosis (Figures 3C,D) and immune processes including antigen processing and presentation, lymphocytes activation, and interleukin secretion (Figures 3E,F and Supplementary Table 6). These results indicated that cluster 1 exhibited high apoptotic and immune characteristics.

### Cluster 1 Had Low Tumor Purity and High Immune Infiltration

**FIGURE 4:**
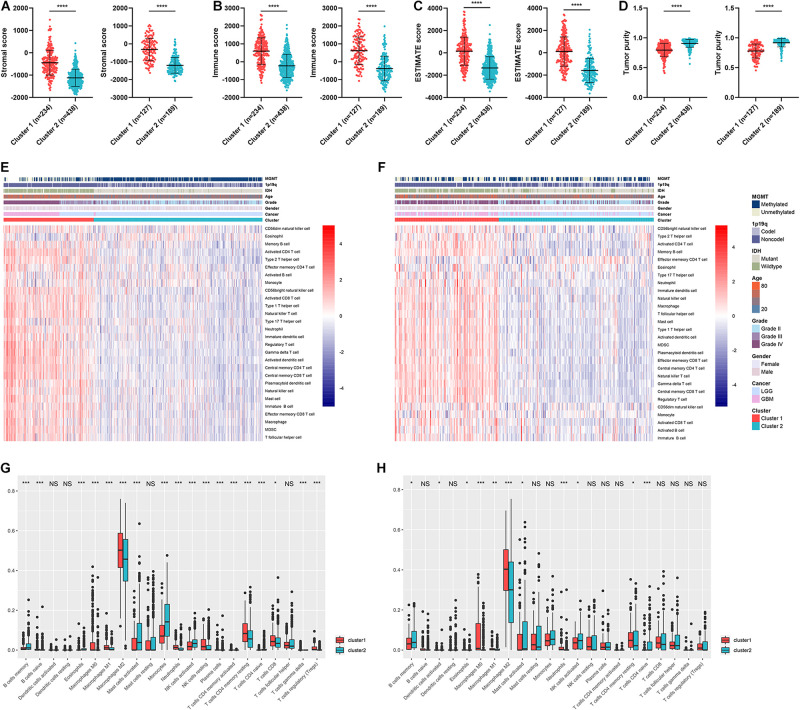
Tumor purity and immune infiltration in two clusters. **(A–D)** The stromal score **(A)**, immune score **(B)**, ESTIMATE score **(C)**, and tumor purity **(D)** of two clusters in TCGA and CGGA datasets. **(E,F)** The infiltration of different immune cells revealed by ssGSEA algorithm in two clusters in TCGA **(E)** and CGGA **(F)** datasets. **(G,H)** CIBERSORT algorithm revealed the abundance of different immune cells in two clusters in TCGA **(G)** and CGGA **(H)** datasets. ^∗^*p* < 0.05; ^∗∗^*p* < 0.01; ^∗∗∗^*p* < 0.001; ^****^p < 0.0001.

Given the fact that cluster 1 was associated with immune processes and poor prognosis, we further explore the differences of immune infiltration between the two clusters. The stromal and immune scores were calculated based on the expression of relevant genes of each sample, and they were significantly elevated in cluster 1 (*p* < 0.05) (Figures 4A,B). Similarly, the ESTIMATE score was significantly higher in cluster 1 compared with cluster 2 (*p* < 0.05) (Figure 4C). Tumor purity was calculated based on the ESTIMATE score, which was significantly lower in cluster 1 (*p* < 0.05) (Figure 4D). Since low tumor purity indicated high immune infiltration, we conducted ssGSEA and CIBERSORT algorithms to quantify the abundance of different immune cells. Results showed that immune cells such as macrophages, neutrophils, activated CD4^+^ T cells, and activated dendritic cells were enriched in cluster 1 (Figures 4E,F). Similarly, CIBERSORT revealed that the abundance of macrophage, neutrophils, activated natural killer (NK) cells, and CD4^+^ T cells were significantly elevated in cluster 1 whereas memory B cells were significantly increased in cluster 2 (*p* < 0.05) (Figures 4G,H). These results verified that clustering based on ARL expression revealed a distinct subtype of gliomas, which was associated with poor prognosis, activated immune processes, and high immune infiltration.

### ARL Signature Correlated With Prognosis and Clinical Features of Gliomas

**FIGURE 5:**
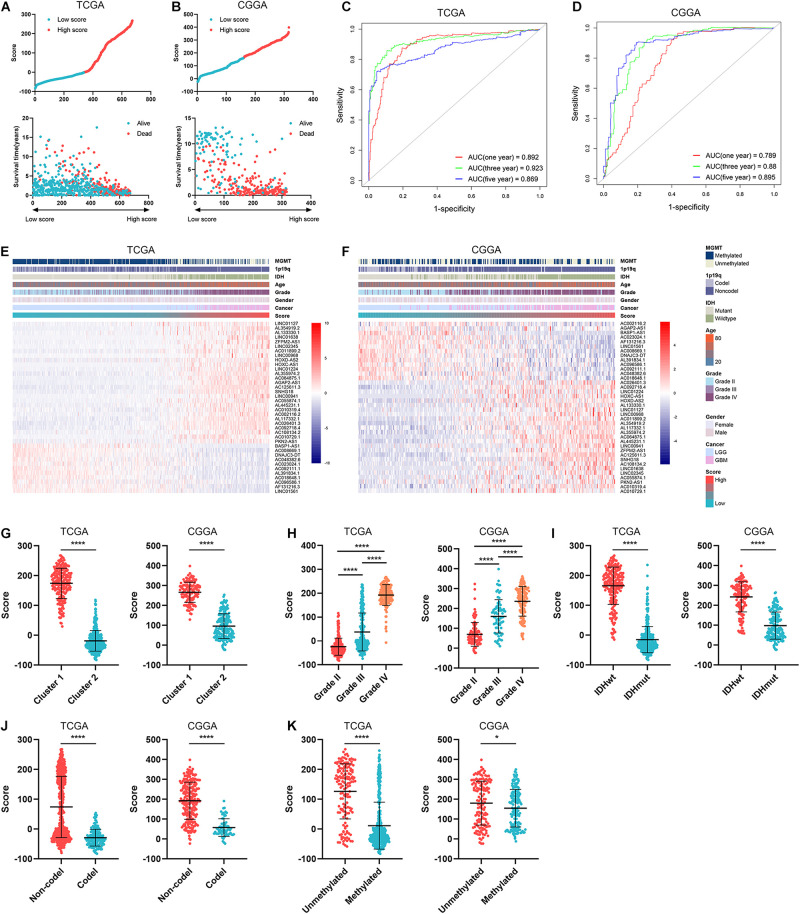
ARL signature correlated with prognosis and clinical features of gliomas. **(A,B)** ARL score was calculated in diffuse glioma samples in TCGA **(A)** and CGGA **(B)** datasets. **(C,D)** Time-dependent ROC analysis of ARL score in predicting the survival of glioma patients in TCGA **(C)** and CGGA **(D)** datasets. **(E,F)** The expression of 38 ARLs in low-score and high-score samples in TCGA **(E)** and CGGA **(F)** datasets. **(G)** ARL score in two clusters in TCGA and CGGA datasets. **(H–K)** ARL score in different grades **(H)**, IDH1 status **(I)**, MGMT status **(J)**, and 1p19q status **(K)** of gliomas in in TCGA and CGGA datasets. ^∗^*p* < 0.05; ^****^p < 0.0001.

To further explore the role of ARLs in gliomas, we constructed an ARL signature and calculated the ARL score of each sample by PCA analysis. Glioma patients were divided into high-score and low-score groups with the cutoff point of median ARL score, and they were well classified based on ARL score (Figures 5A,B and Supplementary Figure 3). Patients with relatively short survival time and censored were enriched in the high-score group (Figures 5A,B). Besides, the ARL score exhibited potent predictive accuracy in the prognosis of glioma patients. The area under curve (AUC) of ARL score in predicting 1-, 3-, and 5-year survival was 0.892, 0.923, and 0.869, respectively, in the TCGA dataset (Figure 5C); that in the CGGA dataset was 0.789, 0.880, and 0.895, respectively (Figure 5D). Meanwhile, 11 protective ARLs were highly expressed in the low-score group whereas 27 risk ARLs were highly expressed in the high-score group (Figures 5E,F). As for clinical features, ARL score was significantly elevated in cluster 1, which was associated with poor prognosis (*p* < 0.05) (Figure 5G). Moreover, ARL score was significantly increased in a grade-dependent manner, which was highest in grade IV glioma (*p* < 0.05) (Figure 5H). Additionally, ARL score was significantly higher in *IDH1* wildtype, 1p19q non-codeleted, and MGMT unmethylated gliomas (*p* < 0.05) (Figures 5I–K). These findings suggested that ARL signature could predict the prognosis of glioma patients and the high ARL score indicated a malignant subtype of gliomas.

### ARL Score Indicated the Prognosis in Different Subgroups of Glioma Patients

**FIGURE 6:**
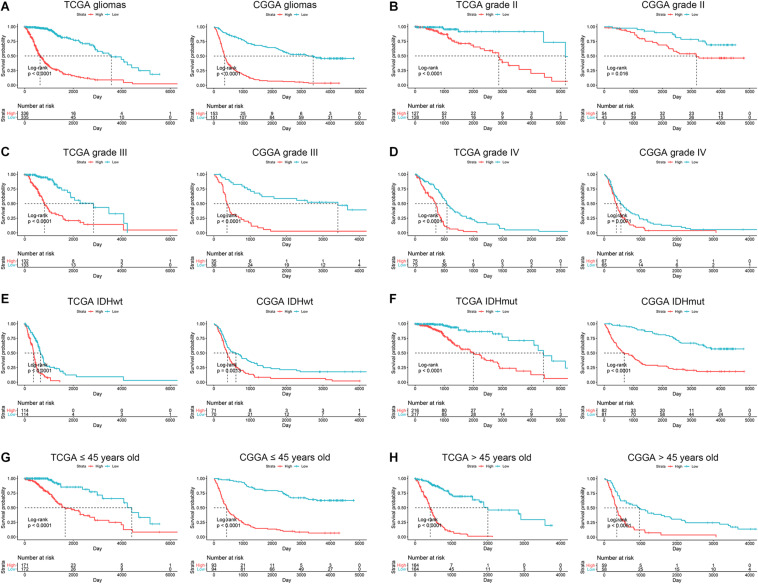
ARL score indicated the prognosis in different subgroups of glioma patients. **(A)** Kaplan-Meier analysis of high-score and low-score patients in diffuse gliomas. **(B–D)** Kaplan-Meier analysis of high-score and low-score patients in grade II **(B)**, grade III **(C)**, and grade IV **(D)** gliomas. **(E,F)** Kaplan-Meier analysis of high-score and low-score patients in IDH1 wildtype **(E)** and IDH1 mutant **(F)** gliomas. **(G,H)** Kaplan-Meier analysis of high-score and low-score patients ≤45 years old **(G)** or >45 years old **(H)** in diffuse gliomas.

Since ARL score exhibited potent accuracy in predicting the survival of glioma patients, we further explore the correlation between ARL score and prognosis in different subgroups of glioma patients. Multivariate Cox analysis revealed that ARL score, age, grade, and *IDH1* status were independent protective factors for glioma patients (Table 2). Besides, the stratification based on ARL score could efficiently distinguish glioma patients with favorable or unfavorable prognoses. In diffuse gliomas, patients with low ARL score had a longer survival time compared with those with high ARL score (*p* < 0.05) (Figure 6A). In different grades of gliomas especially in grade IV glioma, patients with low ARL score had significantly favorable prognoses (*p* < 0.05) (Figures 6B–D). Moreover, in *IDH1* wildtype and mutant gliomas, low ARL score indicated a better prognosis than those with high ARL score (*p* < 0.05) (Figures 6E,F). Similarly, in young (≤45 years old) and old (>45 years old) glioma patients, those with low ARL score had more favorable prognoses compared with those with high ARL score (*p* < 0.05) (Figures 6G,H). Therefore, ARL signature was associated with the prognosis of glioma patients, and low ARL score indicated a favorable prognosis.

**TABLE 2:** Multivariate analysis of ARL score in TCGA and CGGA datasets.

Variables	TCGA (*n* = 672)		CGGA (*n* = 316)	
		
	HR (95%CI)	*P*-value	HR (95%CI)	*P*-value
ARL score	1.016 (1.012–1.020)	<0.001	1.007 (1.004–1.009)	<0.001
Age	1.031 (1.018–1.044)	<0.001	1.013 (1.000–1.027)	0.045
Grade	1.173 (1.010–1.336)	<0.001	1.874 (1.487–2.361)	<0.001
IDH1	0.514 (0.293–0.902)	0.020	0.402 (0.247–0.652)	<0.001
1p19q	1.362 (0.805–2.304)	0.250	2.894 (1.683–4.977)	<0.001
MGMT	1.352 (0.974–1.877)	0.072	1.214 (0.892–1.652)	0.218

*HR, hazard ratio; CI, confidence interval; IDH, isocitrate dehydrogenase; MGMT, O6-methylguanine-DNA methyltransferase.*

### ARL Score Was Associated With Immune Infiltration in Diffuse Gliomas

**FIGURE 7:**
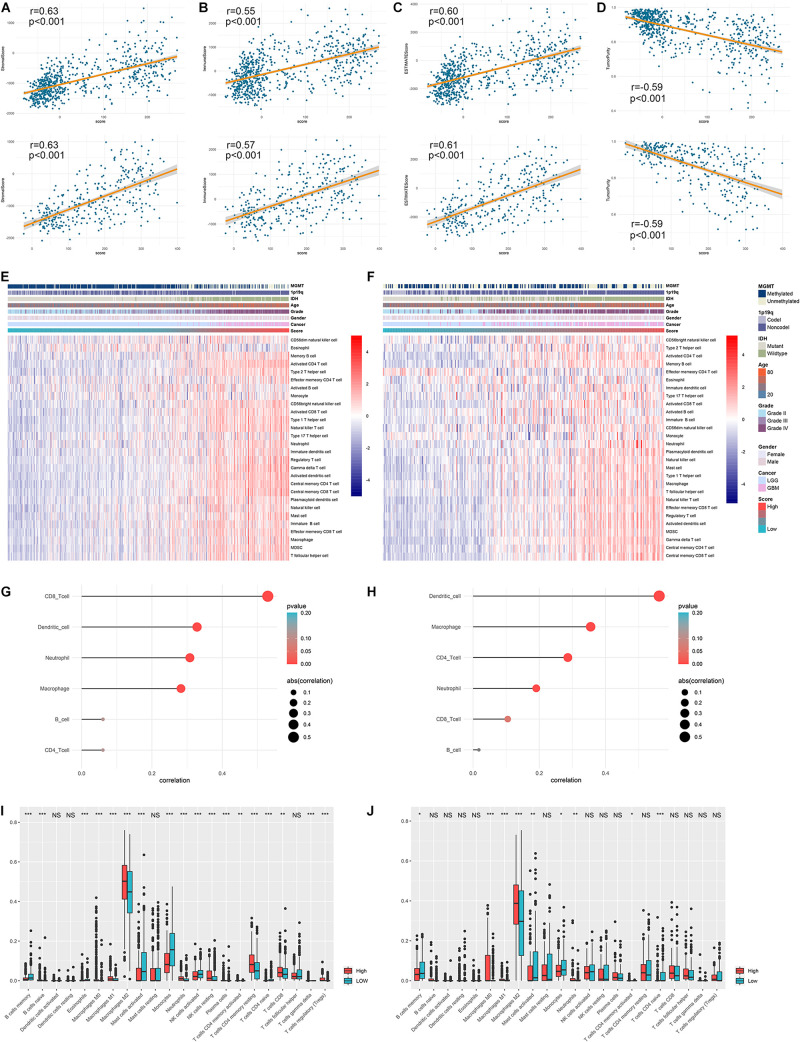
ARL score was associated with immune infiltration in diffuse gliomas. **(A–D)** Correlation between ARL score with stromal score **(A)**, immune score **(B)**, ESTIMATE score **(C)**, and tumor purity **(D)** in TCGA and CGGA datasets. **(E,F)** The infiltration of different immune cells revealed by ssGSEA algorithm in low-score and high-score patients in TCGA **(E)** and CGGA **(F)** datasets. **(G,H)** The correlation between ARL score with the abundance of six immune cells estimated by TIMER algorithm in TCGA **(G)** and CGGA **(H)** datasets. **(I,J)** CIBERSORT algorithm revealed the abundance of different immune cells in low-score and high-score groups in TCGA **(I)** and CGGA **(J)** datasets. ^∗^*p* < 0.05; ^∗∗^*p* < 0.01; ^∗∗∗^*p* < 0.001.

Considering that ARL score was significantly elevated in cluster 1 and cluster 1 exhibited high immune infiltration, we further investigated the correlation between ARL score and immune infiltration in diffuse gliomas. By the conduct of Pearson correlation analysis, we found that ARL score was significantly positively correlated with the stromal score, immune score, and ESITMATE score (*p* < 0.05) (Figures 7A–C). Besides, ARL score was significantly negatively correlated with tumor purity (*p* < 0.05); (Figure 7D). In high-score group, ssGSEA algorithm revealed that NK cells, neutrophils, macrophages, activated CD4^+^ T cells, and activated dendritic cells were highly enriched (Figures 7E,F). Moreover, ARL score was significantly positively correlated with the abundance of macrophages, dendritic cells, and neutrophils, which was revealed by TIMER algorithm (*p* < 0.05) (Figures 7G,H). Additionally, in CIBERSORT algorithm, the infiltration of macrophages and neutrophils was significantly higher in high-score group compared with low-score group (*p* < 0.05) (Figures 7I,J). These results suggested that ARL score was associated with immune infiltration and high ARL score indicated high infiltration of macrophages and neutrophils in diffuse gliomas.

## Discussion

Tumor adaptations were necessary for its progression, in which autophagy mediated diverse cellular reactions and played a crucial role in gliomas (Ulasov et al., 2018). In this study, we applied an unsupervised clustering method to reveal a novel subtype of gliomas, which was associated with the prognosis and had distinct clinical features between two clusters. Cluster 1, which had a poor prognosis, exhibited enriched apoptotic and immune characteristics. Moreover, cluster 1 had a low purity and high immune infiltration. The constructed ARL signature had a potent accuracy in predicting the prognosis of glioma patients. ARL score was significantly elevated in the malignant subtype of glioma and high ARL score indicated a poor prognosis. Besides, high ARL score was significantly correlated with low tumor purity and high immune infiltration. Our study provided novel insights into the prognostic value of ARL and its correlation with immune microenvironment in diffuse gliomas.

Based on the expression of different genes, cancers can be classified into different subtypes, which exhibited different malignancies and clinical outcomes. In head and neck cancer, 54 hypoxia and immune genes can divide patients into three groups: low-hypoxia/high-immune, high-hypoxia/low-immune, and mixed, in which low-hypoxia/high-immune group had the most favorable prognosis with the 5-year OS rate of 71% (Brooks et al., 2019). Similarly, low-hypoxia/high-immune group of triple-negative breast cancer had a relatively favorable survival time (Zheng et al., 2020). Moreover, gliomas were classified into two subgroups based on the expression of epithelial-mesenchymal transition (EMT) genes, in which EM1 subgroup had a better prognosis and EM2 subgroup was associated with cancer development and progression (Tao et al., 2020). In our study, we identified a unique subtype of gliomas based on the expression of ARLs, in which cluster 1 was associated with poor prognosis and enriched with the malignant subtype of gliomas including *IDH1* wildtype, 1p19q non-codeleted, and MGMT unmethylated ones. These results indicated that ARL could be potential biomarkers to distinguish a malignant subtype from others in gliomas. Moreover, cluster 1 exhibited distinct high apoptotic and immune characteristics. Previous studies suggested that tumors were adaptive to reduce apoptosis and induce immune evasion (Wong, 2011; Vinay et al., 2015). The high apoptotic and immune characteristics might facilitate the development and progression of malignant subtypes of gliomas. Additionally, tumor purity was associated with clinical and molecular subtypes of gliomas; low tumor purity indicated malignant subtype of glioma and poor prognosis of glioma patients (Zhang C. et al., 2017). In our study, cluster 1, the subgroup with poor prognoses, had significantly reduced tumor purity and high immune infiltration, which was consistent with previous findings. Therefore, we classified diffuse gliomas into two clusters based on the expression of ARLs, which was associated with the prognosis and immune microenvironment of diffuse gliomas.

Luan et al. (2019) have identified 402 ARLs in CGGA microarray dataset with the cutoff value of | R| > 0.3 and *p* < 0.05, where 10 prognostic ARLs were selected to construct the ARL signature. In our study, 2,539 ARLs were screened in the TCGA and CGGA datasets with the cutoff value of | R| > 0.5 and *p* < 0.01, in which six ARLs (*ZNF674-AS1, MAPKAPK5-AS1, COX10-AS1, GABPB1-AS1, DDX11-AS1, SBF2-AS1*, and *MIR4453HG*) were identified as ARLs before (Luan et al., 2019). Compared with the previous ARL signature, our study constructed a novel 38-ARL signature based on PCA algorithm, which was different from the previous 10-ARL signature. Besides, we explored the predictive accuracy of ARL score regarding the OS of glioma patients and performed subgroup analysis to explicitly validate the prognostic value of ARL signature in two independent datasets. Moreover, our study firstly classified gliomas into two subtypes based on the expression of ARLs, which was significantly associated with the prognosis and immune microenvironment of gliomas.

Among identified ARLs, several ARLs have been reported to play crucial roles in various cancers. Previous studies indicated that *LINC01224* could promote the proliferation and invasion of epithelial ovarian cancer, gastric cancer, and hepatocellular carcinoma (Gong et al., 2020; Xing et al., 2020; Sun et al., 2021). *SNHG18* promoted the radioresistance of glioma cells and the metastasis of non-small cell lung cancer (Zheng et al., 2016; Fan et al., 2021). *ZFPM2-AS1* could promote the development of gastric cancer, lung adenocarcinoma, and hepatocellular carcinoma (Kong et al., 2018; He et al., 2020; Xue et al., 2020). Meanwhile, these ARLs were associated with the prognosis of cancer patients. Therefore, the prognostic value of ARLs exhibited not only in gliomas but also in other cancers.

The construction of gene signature has shown a promising ability in predicting the prognosis of cancer patients. Our previous studies revealed that immune-related genes and N6-methylandenosine (m6A) regulators were associated with the prognosis of glioma patients (Xu et al., 2020a, 2021). Liang et al. (2020) found that ferroptosis-related genes were differentially expressed in hepatocellular carcinoma (HCC), and ferroptosis-related signature was an independent predictor for HCC patients. Similarly, a ferroptosis-related signature was correlated with clinical and pathological features of glioma (Liu H.J. et al., 2020). Immune-related lncRNAs were associated with the prognosis and immune landscape of HCC patients (Hong et al., 2020). In our study, we constructed an ARL signature using PCA algorithm. ARL score was significantly elevated in cluster 1 and grade IV gliomas. Moreover, ARL score was significantly increased in the relatively malignant subtype of gliomas such as *IDH1* wildtype, 1p19q non-codeleted, and MGMT unmethylated gliomas. In routine prognostic indicators such as age, grade, *IDH1*, MGMT, and 1p19q status, tumor grade had the highest AUC in predicting the survival of glioma patients (Supplementary Figure 5). In contrast, the AUC of ARL score in predicting the prognosis of glioma patients was higher than that of tumor grade and other indicators. Besides, although current indicators could predict the prognosis of glioma patients, our study revealed that low ARL score was associated with favorable prognosis in different subgroups of glioma patients. Therefore, the ARL signature could further stratify patients as supplementary to current indicators.

Tumor immune microenvironment has attracted more and more attention due to its crucial role in cancer progression (Quail and Joyce, 2017; Wang et al., 2020; Zhang X. et al., 2020; Zhang Y. et al., 2020). Previous studies indicated that lncRNAs could affect tumor immune microenvironment and thus promote tumor progression (Xu et al., 2019; Sun et al., 2020). Autophagy could modulate the immunosuppressive context of glioma cells, which facilitate their progression (Molina et al., 2019). Meanwhile, autophagy-related genes were demonstrated to correlate with the prognosis and immune microenvironment of gliomas (Xu Y. et al., 2020). However, the association between ARLs and immune microenvironment remained unclear. The immune microenvironment of glioma was characterized by an immunosuppressive status. Glioma cells could stimulate the transition of TAMs from M1 to M2 subtype in their context, which facilitated the immune evasion of glioma cells and promote their proliferation (Badie and Schartner, 2000). Besides, tumor-associated neutrophils (TANs) played a diverse role in glioma microenvironment. Previous studies suggested that TANs mediated cytotoxic activities against tumor cells and suppress metastasis (Granot et al., 2011; Shaul and Fridlender, 2019). In contrast, TANs could promote tumor progression by stimulating angiogenesis and suppressed the activity of CD8^+^ T cells (Antonio et al., 2015; Bodogai et al., 2015). However, the high circulating TANs remained to be a robust biomarker of unfavorable prognosis in various cancers (Shaul and Fridlender, 2019). In our study, we found that ARL score was negatively associated with tumor purity. Therefore, high ARL score indicated low tumor purity and high immune infiltration. By performing several algorithms to characterize the abundance of different immune cells in glioma samples, we found that macrophages and neutrophils were highly enriched in high ARL score group, and their abundance was significantly positively associated with ARL score. Especially, the abundance of M2 subtype of macrophage was significantly higher in high-score group than low-score group. Since M2 macrophage mediated immunosuppressive context, its elevation would facilitate the progression of glioma cells (Mantovani et al., 2002), which accounted for the high malignancy and poor prognosis of gliomas in high-score group.

There are still some limitations in our study. Firstly, our findings are mainly concluded based on online databases. The verification of an independent cohort will further validate our findings. Besides, the molecular mechanism underlying ARLs in the development and progression of glioma cells remains unclear, which requires further investigation. However, our study still reveals novel subtypes of diffuse glioma and uncovers the correlation between ARLs and immune microenvironment in gliomas.

## Conclusion

To sum up, our study developed and validated a novel ARL signature for the classification of diffuse glioma, which was closely associated with glioma immune microenvironment and could serve as a promising prognostic biomarker for glioma patients.

## Data Availability Statement

Publicly available datasets were analyzed in this study. This data can be found here: The data are accessible in TCGA (https://portal.gdc.cancer.gov/) and CGGA (http://www.cgga.org.cn/) databases.

## Author Contributions

QC and KY conceived, designed, and supervised the study. SX drafted the manuscript and performed data analysis and visualization. SX, LT, and ZL collected the data. All authors reviewed and approved the final manuscript.

## Conflict of Interest

The authors declare that the research was conducted in the absence of any commercial or financial relationships that could be construed as a potential conflict of interest.

## Footnotes

1 https://portal.gdc.cancer.gov/

2 http://www.cgga.org.cn/

3 http://www.autophagy.lu/

## Supplementary Material

Supplementary Figure 1 LASSO regression analysis of ARLs in TCGA dataset.

Supplementary Figure 2 Kaplan-Meier analysis of randomly selected eight ARLs in TCGA and CGGA datasets.

Supplementary Figure 3 Volcano plot of differentially expressed genes between cluster 1 and cluster 2 in TCGA and CGGA datasets.

Supplementary Figure 4 PCA analysis of stratification of glioma patients based on ARL score.

Supplementary Figure 5 Time-dependent ROC analysis of different clinical features of gliomas.

## Abbreviations

OS: overall survival
IDH: isocitrate dehydrogenase
MGMT: O6-methylguanine-DNA methyltransferase
CQ: chloroquine
HCQ: hydroxychloroquine
lncRNA: long non-coding RNA
TMZ: temozolomide
ARLs: autophagy-related lncRNAs
TCGA: The Cancer Genome Atlas
CGGA: Chinese Glioma Genome Atlas
GSVA: gene set variation analysis
GSEA: gene set enrichment analysis
FDR: false discovery rate
GO: gene Ontology
ESTIMATE: Estimation of Stromal and Immune cells in Malignant Tumor tissues using Expression data
ssGSEA: single-sample Gene Set Enrichment Analysis
TIMER: Tumor Immune Estimation Resource
LASSO: Least Absolute Shrinkage and Selection Operator
PCA: principal component analysis
ROC: receiver operating characteristic
EMT: epithelial-mesenchymal transition
HCC: hepatocellular carcinoma
TAMs: tumor-associated macrophages
Treg: regulatory T cells
TANs: tumor-associated neutrophils.
