# Supplementary figures and images for: Bioinformatic Analyses Identify a Prognostic Autophagy-Related Long Non-coding RNA Signature Associated With Immune Microenvironment in Diffuse Gliomas
Source: Front Cell Dev Biol. 2021 Jun 15;9:694633. doi: 10.3389/fcell.2021.694633 (PMC8239411; doi:10.3389/fcell.2021.694633)

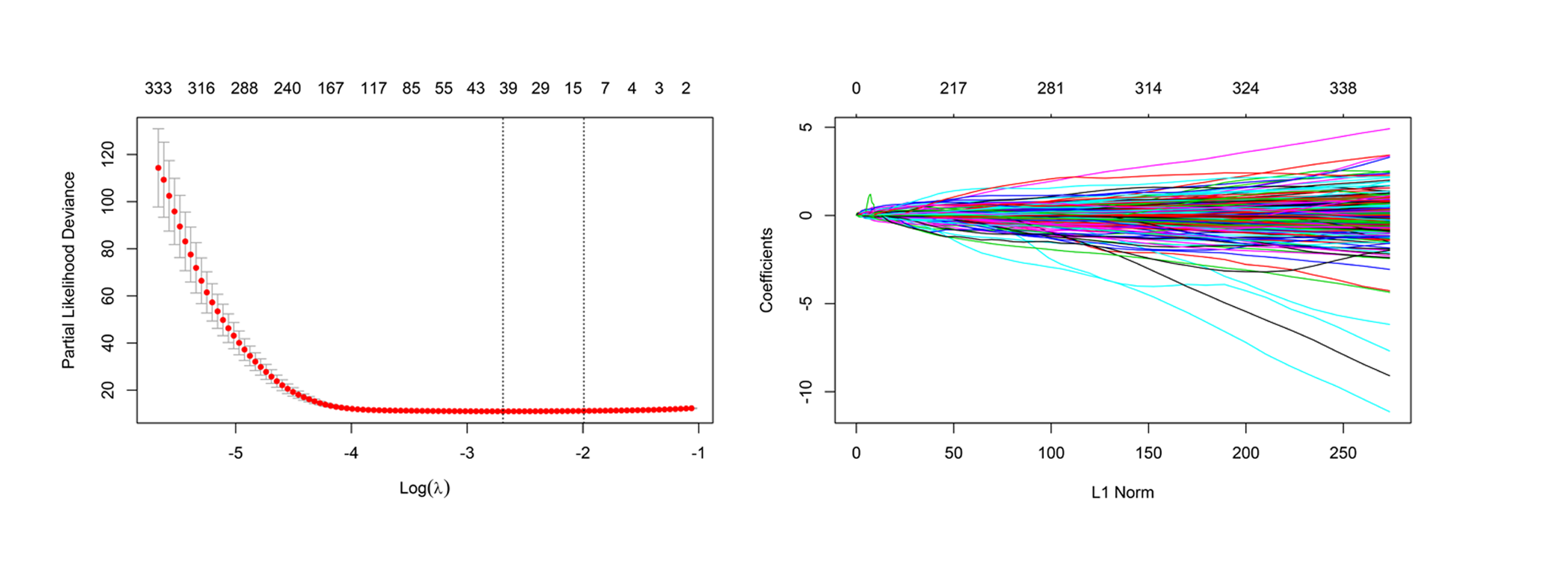

Supplement: Supplementary Figure 1 — LASSO regression analysis of ARLs in TCGA dataset. [file Image_1.TIF]

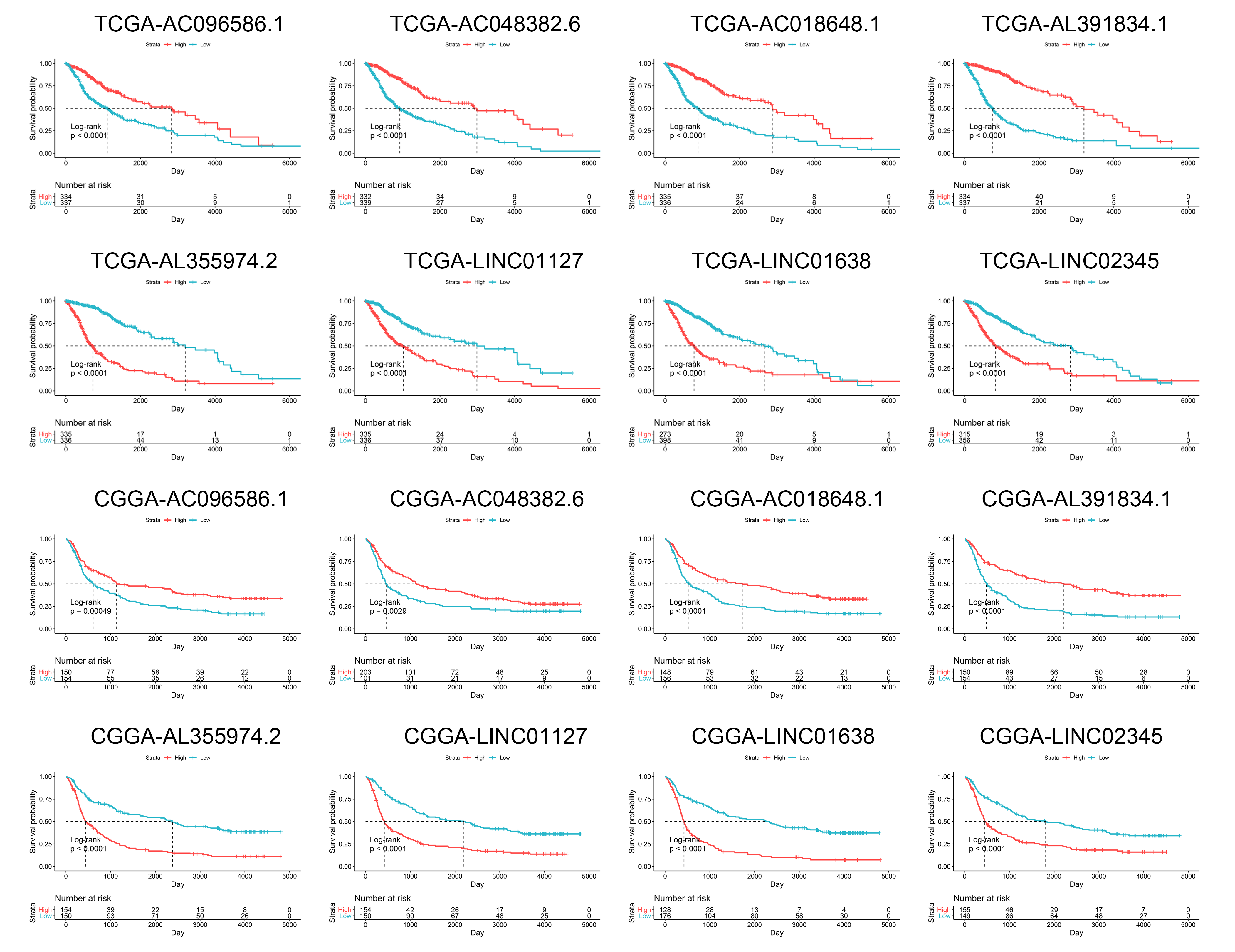

Supplement: Supplementary Figure 2 — Kaplan-Meier analysis of randomly selected eight ARLs in TCGA and CGGA datasets. [file Image_2.TIF]

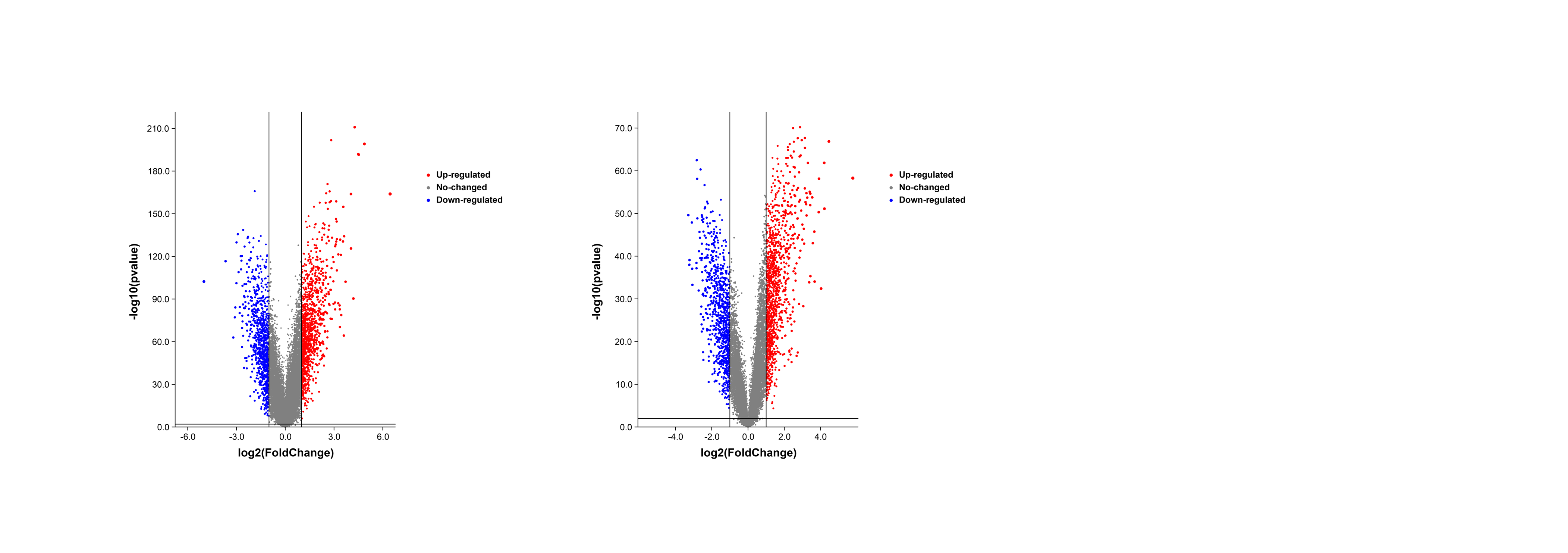

Supplement: Supplementary Figure 3 — Volcano plot of differentially expressed genes between cluster 1 and cluster 2 in TCGA and CGGA datasets. [file Image_3.TIF]

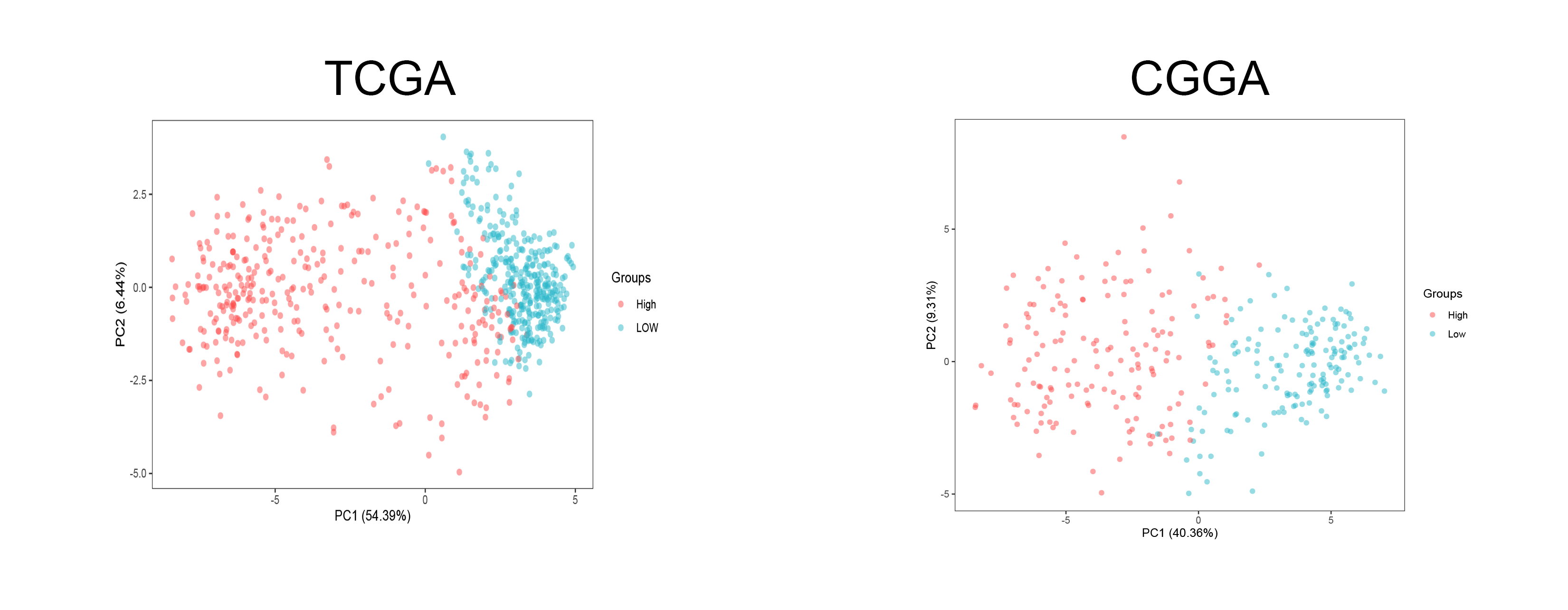

Supplement: Supplementary Figure 4 — PCA analysis of stratification of glioma patients based on ARL score. [file Image_4.TIF]

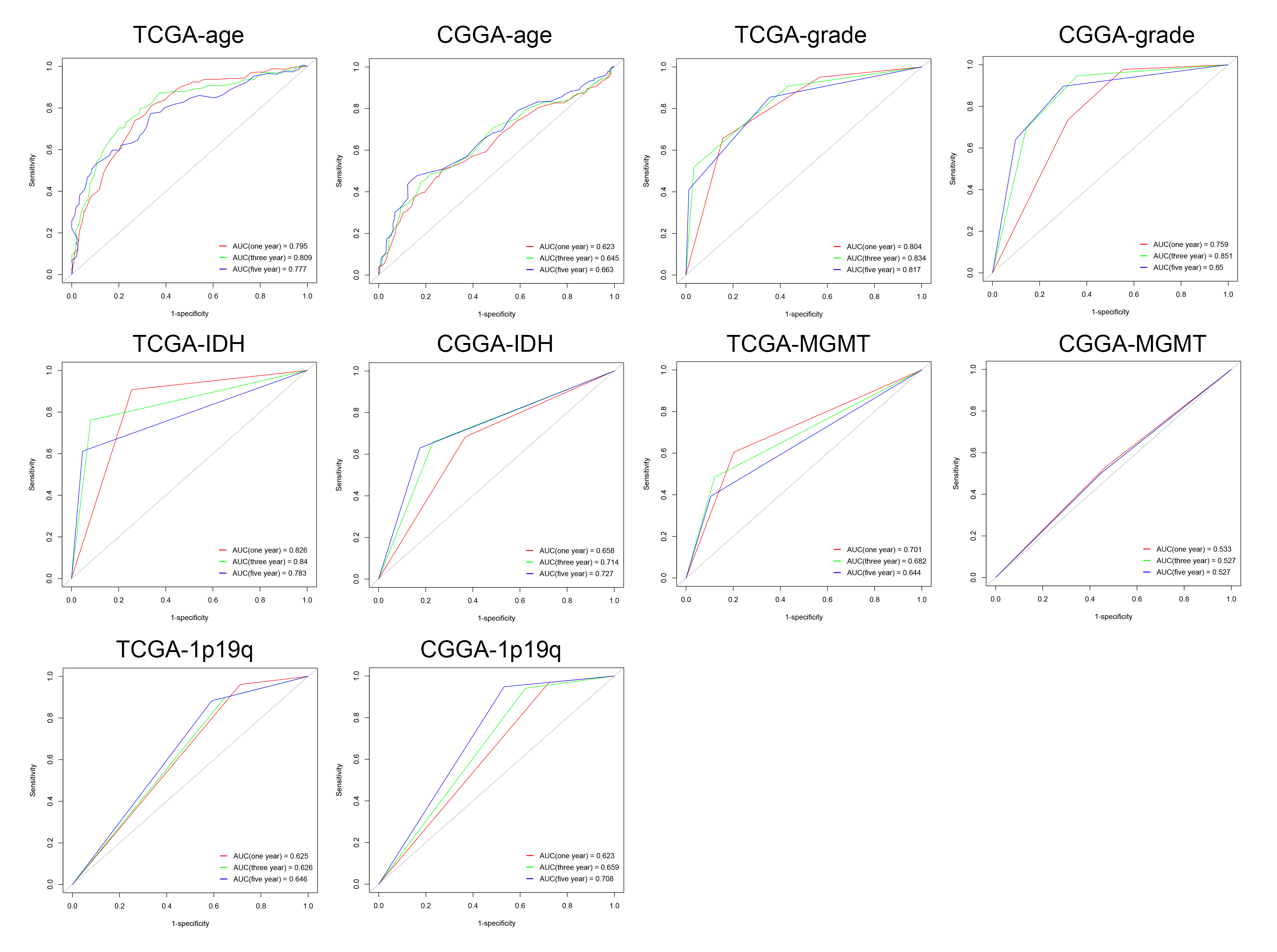

Supplement: Supplementary Figure 5 — Time-dependent ROC analysis of different clinical features of gliomas. [file Image_5.TIF]
